# Supplementary material for: Galantamine attenuates autoinflammation in a mouse model of familial mediterranean fever
Source: Mol Med. 2022 Dec 9;28:148. doi: 10.1186/s10020-022-00571-9 (PMC9733251; doi:10.1186/s10020-022-00571-9)
Supplement: Supplementary file 1 — Additional file 1: Figure S1. (A) Weights (mg) of FMF-KI spleens used for flow cytometric analysis after 4-8 weeks of treatement with galantamine or PBS. (B) Example flow cytometric analysis of splenic and peritoneal cells after 4-8 weeks of treatment. CD11b+ cells were analyzed for relative frequencies of Ly6G+ neutrophils and F4/80+ macrophages. The percentage of neutrophils in CD11b+ cells from the spleens and peritonea of galantamine- and PBS-treated FMF-KI mice (n = 5 in each group) is shown next to the flowcytometry panels. (C) Spleen weights (mg) from galantamine- and PBS-treated mice after 6 days of treatment with galantamine (twice daily, escalated to 12 mg/kg). Data presented as mean ± SEM, P by Mann-Whitney U test. Figure S2. Serum cytokine levels from FMF-KI mice after 8 weeks of treatment with galantamine. (A and B) IL-6 (A), IL-1β, INF-γ, TNF-α, and IL-10 (B) levels in sera from FMF mice measured by multiplex ELISA (data presented as mean ± SEM, P value by Mann-Whitney U test compared to PBS). Figure S3. (A) IL-1β release measured by ELISA from FMF-KI BMDM primed with LPS and treated with Ach (with 100 mM pyridostigmine) and colchicine (left panel) or nicotine (right panel). Data presented as mean ± SD from duplicate measurement, *P <0.05 by ANOVA. [file 10020_2022_571_MOESM1_ESM.docx]

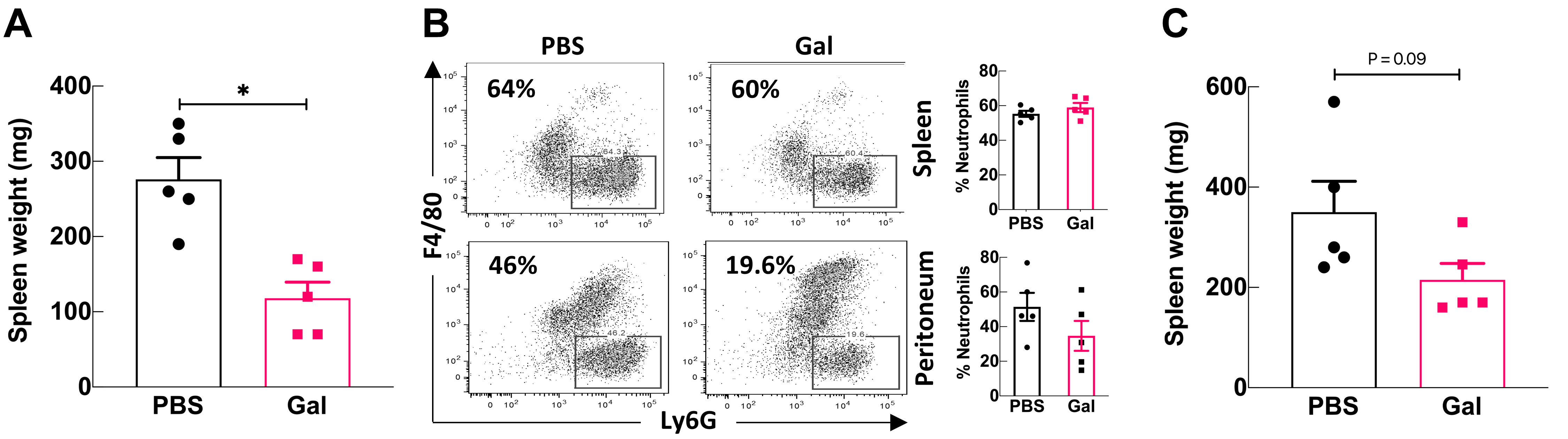


**Figure S 1. (A)** Weights (mg) of FMF-KI spleens used for flow cytometric analysis after 4-8 weeks of treatement with galantamine or PBS. **(B)** Example flow cytometric analysis of splenic and peritoneal cells after 4-8 weeks of treatment. CD11b^+^ cells were analyzed for relative frequencies of Ly6G^+^ neutrophils and F4/80^+^ macrophages. The percentage of neutrophils in CD11b^+^ cells from the spleens and peritonea of galantamine- and PBS-treated FMF-KI mice (n = 5 in each group) is shown next to the flowcytometry panels. **(C)** Spleen weights (mg) from galantamine- and PBS-treated mice after 6 days of treatment with galantamine (twice daily, escalated to 12 mg/kg). Data presented as mean ± SEM, *P* by Mann-Whitney U test.


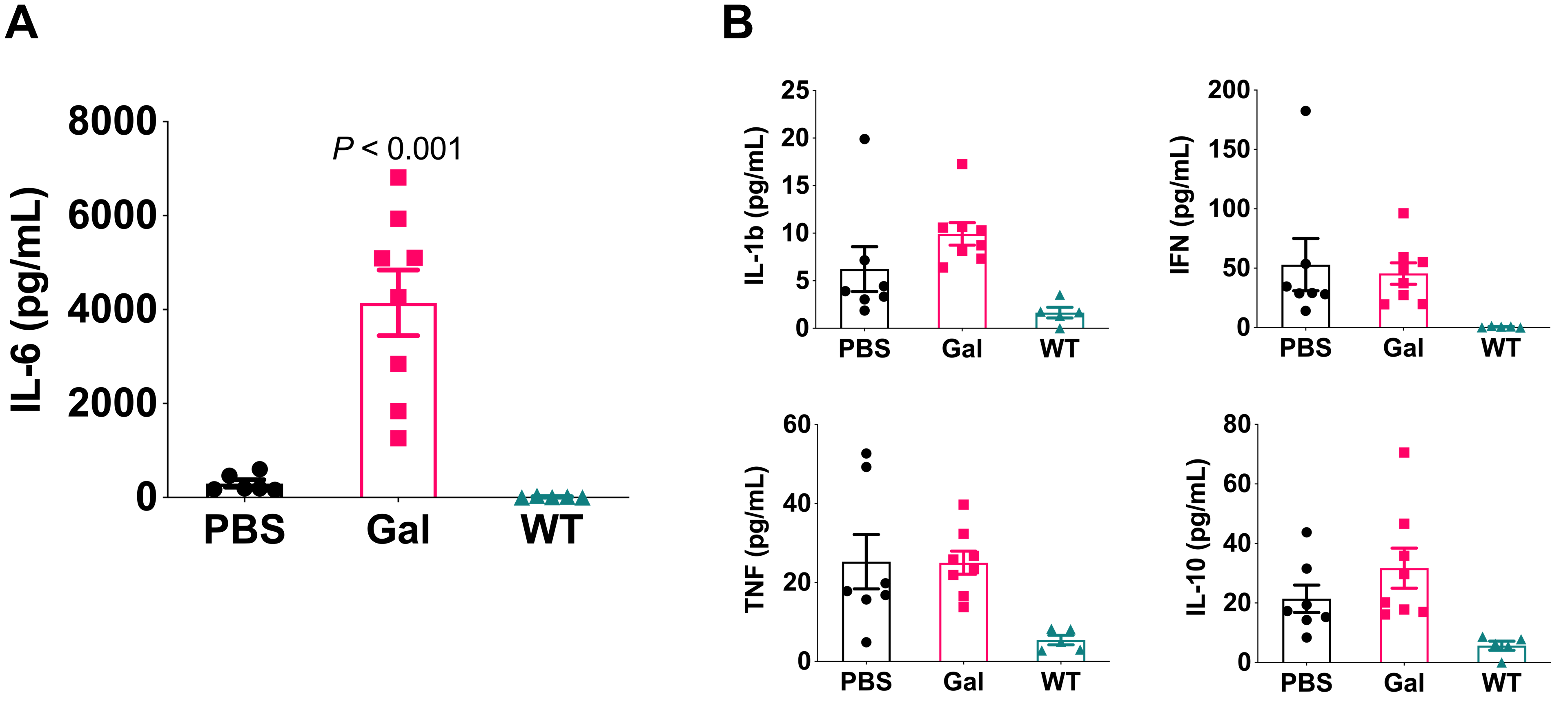


**Figure S 2. Serum cytokine levels from FMF-KI mice after 8 weeks of treatment with galantamine.** **(A and B)** IL-6 (A), IL-1β, INF-γ, TNF-α, and IL-10 (B) levels in sera from FMF mice measured by multiplex ELISA (data presented as mean ± SEM, *P* value by Mann-Whitney U test compared to PBS).


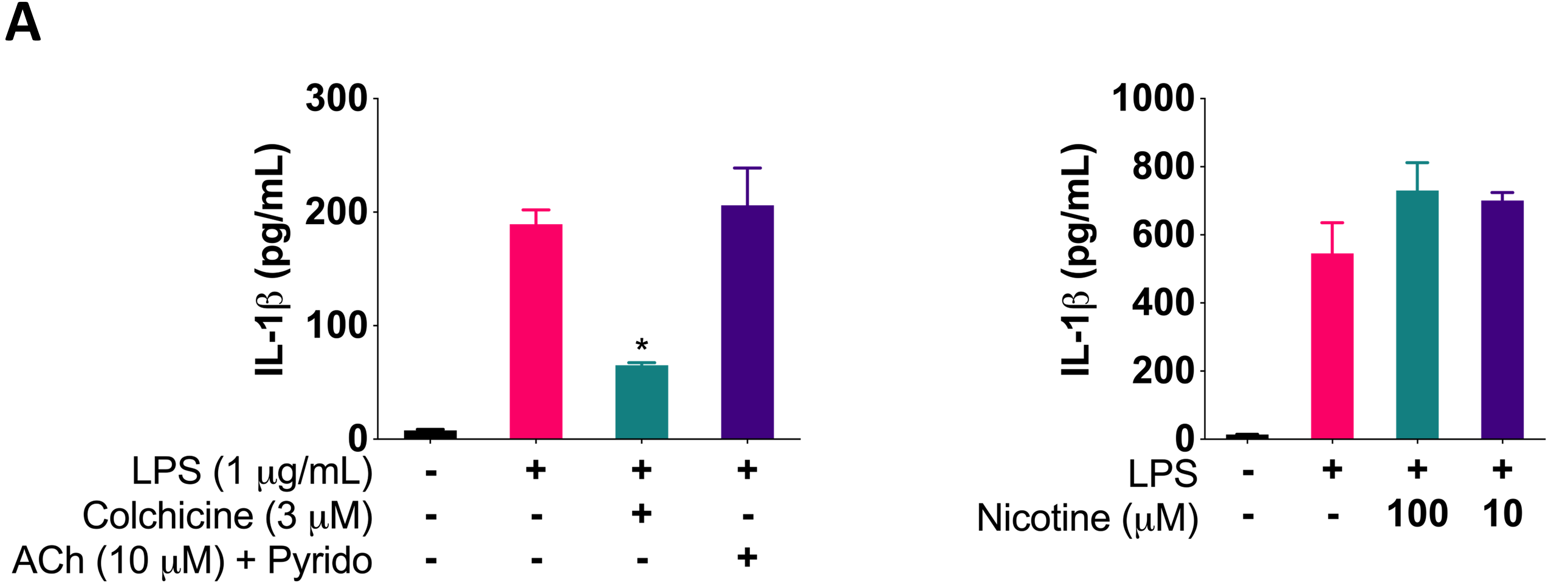


**Figure S 3. (A)** IL-1β release measured by ELISA from FMF-KI BMDM primed with LPS and treated with Ach (with 100 mM pyridostigmine) and colchicine (left panel) or nicotine (right panel). Data presented as mean ± SD from duplicate measurement, **P* <0.05 by ANOVA.
